# High-throughput proteomics integrated with gene microarray for discovery of colorectal cancer potential biomarkers

**DOI:** 10.18632/oncotarget.12143

**Published:** 2016-09-20

**Authors:** Jiekai Yu, Xiaofen Li, Chenhan Zhong, Dan Li, Xiaohui Zhai, Wangxiong Hu, Cheng Guo, Ying Yuan, Shu Zheng

**Affiliations:** ^1^ Cancer Institute (Key Laboratory of Cancer Prevention and Intervention, China National Ministry of Education), the Second Affiliated Hospital, Zhejiang University School of Medicine, China; ^2^ Department of Medical Oncology, The Second Affiliated Hospital, Zhejiang University School of Medicine, Zhejiang, China

**Keywords:** iTRAQ-MS, gene microarray, colorectal cancer, biomarkers

## Abstract

Proteins, as executives of genes' instructions, are responsible for cellular phenotypes. Integrating proteomics with gene microarray, we conducted this study to identify potential protein biomarkers of colorectal cancer (CRC). Isobaric tags with related and absolute quantitation (iTRAQ) labeling mass spectrometry (MS) was applied to screen and identify differentially expressed proteins between paired CRC and adjacent normal mucosa. Meanwhile, Affymetrix U133plus2.0 microarrays were used to perform gene microarray analysis. Verification experiments included immunohistochemistry (IHC), western blot and enzyme-linked immunosorbent assay (ELISA) of selected proteins. Overall, 5469 differentially expressed proteins were detected with iTRAQ-MS from 24 matched CRC and adjacent normal tissues. And gene microarray identified 39859 differential genes from 52 patients. Of these, 3083 differential proteins had corresponding differentially expressed genes, with 245 proteins and their genes showed >1.5-fold change in expression level. Gene ontology enrichment analysis revealed that up-regulated proteins were more involved in cell adhesion and motion than down-regulated proteins. In addition, up-regulated proteins were more likely to be located in nucleus and vesicles. Further verification experiments with IHC confirmed differential expression levels of 5 proteins (S100 calcium-binding protein A9, annexin A3, nicotinamide phosphoribosyltransferase, carboxylesterase 2 and calcium activated chloride channel A1) between CRC and normal tissues. Besides, western blot showed a stepwise increase of annexin A3 abundance in normal colorectal mucosa, adenoma and CRC tissues. ELISA results revealed significantly higher serum levels of S100 calcium-binding protein A9 and annexin A3 in CRC patients than healthy controls, validating diagnostic value of these proteins. Cell experiments showed that inhibition of annexin A3 could suppress CRC cell proliferation and aggressiveness. S100 calcium-binding protein A9, annexin A3, nicotinamide phosphoribosyltransferase, carboxylesterase 2 and calcium activated chloride channel A1 were probably potential biomarkers of colorectal cancer. Annexin A3 was a potentially valuable therapeutic target of CRC.

## INTRODUCTION

According to statistics of World Health Organization (WHO), colorectal cancer (CRC) is the third most common malignancy worldwide, with it being the fourth leading cause of cancer-related deaths [1]. Despite diagnostic and therapeutic advancements, CRC survival rate has hardly changed over the last two decades, with over 50% of patients having regional or distant metastasis at the time of diagnosis [1]. However, 5-year survival rates of patients in different stages vary dramatically, from more than 90% in stage I to less than 10% in stage IV [1, 2]. Additionally, as a heterogeneous disease, almost every patient of CRC has his/her specific features. Therefore, early detection and individual treatment are of great significance for CRC. Screening and identifying new biomarkers are warranted to better diagnose, treat, predict and monitor recurrence of CRC.

In the last decade, the Cancer Genome Atlas (TCGA) has elucidated genomic characteristics of human cancers including glioblastoma [3], ovarian cancer [4], lung cancer [5], breast cancer [6], colorectal cancer [7] and endometrial carcinoma [8]. As executives of genes' instructions, proteins are responsible for cellular phenotypes. Since the existence of post-transcriptional and -translational modifications, cancer proteomics is more complex than genomics. However, so far, there are few integrative analyses of proteomic and genomic data for cancers including CRC, which presents an urgent need to integrate proteomics with genomics to obtain a more comprehensive understanding of bioinformation flow in cancer cells.

In this study, we applied high-throughput proteomics integrated with gene microarray to screen and identify potential biomarkers of CRC. Isobaric tags with related and absolute quantitation (iTRAQ) labeling mass spectrometry (MS) was used as proteomic tool. Meanwhile Affymetrix U133plus2.0 microarrays were applied as gene microarray analysis method. In total, we identified and quantitated 3083 proteins/genes expressed simultaneously in paired colorectal cancer (CRC) and adjacent normal mucosa by iTRAQ-LC-MS/MS and gene microarray, among which 245 proteins showed >1.5-fold change in expression level. In addition, expression levels of 24 proteins amongst the 245 proteins were significantly different (P<0.05) between paired CRC and adjacent normal mucosa independent of cancer stage, which were confirmed in the validation set. Subsequent immunohistochemistry (IHC) experiments validated that S100 calcium-binding protein A9 (S100A9), annexin A3 (ANXA3), nicotinamide phosphoribosyltransferase (NAMPT), carboxylesterase 2 (CES2) and calcium activated chloride channel A1 (CLCA1) were differentially expressed between paired CRC and adjacent normal samples (P<0.05). Besides, using western blotting, we observed that expression level of ANXA3 increased stepwise in normal colorectal mucosa, adenoma and CRC tissues.

## RESULTS

### Differential protein expression profiling with iTRAQ-MS

iTRAQ-MS was used to screen and identify proteins expressed differently between paired CRC and adjacent normal colonic mucosa from 24 patients (Table 1). The median age of the patients was 62.8 years old and 75% were male. Five pooled-samples (stage I, stage II, stage III, stage IV and adjacent normal sample) were obtained and labeled with one of the iTRAQ tags 113, 114, 116, 115, and 117, respectively.

**Table 1a:** Characteristics of tissue samples

Samples application		iTRAQ-MS	Gene microarray	Immunohistochemistry
Median age (years old)		62.8	60.2	62.0
Sex	Male	18	30	9
	Female	6	22	9
TNM stage	I	6	4	2
	II	6	17	6
	III	6	27	9
	IV	6	4	1
Differentiation	High	1	18	3
	Moderate	20	23	14
	Low	3	11	1
Total		24	52	18

**Table 1b:** Characteristics of serum samples

Groups	Median age (years old)	Sex		TNM stage			
		Male	Female	I	II	III	IV
Colorectal cancer patients	61	59	41	12	38	25	25
Healthy controls	50	39	37	-	-	-	-

**Figure 1:**
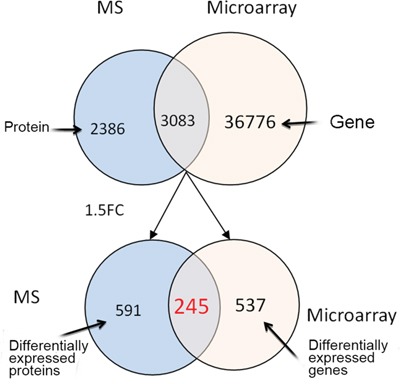
The numbers of differential proteins detected by iTRAQ-MS and genes by gene microarray The blue circles represent proteins identified by iTRAQ-MS, the red circles represent genes identified bygene microarray, and the intersections between blue and red circles contain proteins and their corresponding genes. Abbreviations: MS, mass spectrometry; FC, fold change.

**Figure 2:**
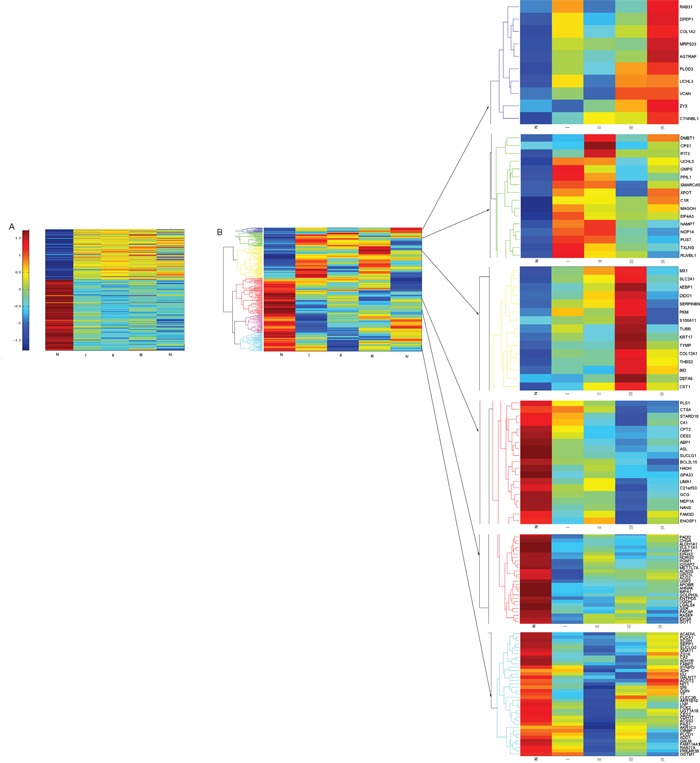
Hierarchical clustering of the 245 proteins/genes expressed significantly different between CRC and normal mucosa Colored scale represents expression ratios between CRC and normal mucosa. Red shades indicate proteins/genes with increased expression, blue shades suggest proteins/genes with reduced expression, and yellow shades indicate minor changes in expression between paired samples. **A.** Hierarchical clustering of the 245 genes expressed significantly different among four stages of CRC and normal mucosa. **B.** Hierarchical clustering of the 245 proteins expressed significantly different among four stages of CRC and normal mucosa. Abbreviation: N, normal colonic mucosa.

In total, 5469 proteins were detected with iTRAQ-MS, including 3104 proteins that had quantitative data. Moreover, 3083 proteins had corresponding expressed genes, which would be elaborated below. By comparing CRC and paired adjacent normal mucosa, there were 836 proteins which showed >1.5-fold change in expression level. In addition, 245 of the 836 proteins had corresponding genes with expression change >1.5-fold. And the regulation directions of 245 proteins were in accordance with their genes thus became our key study objects (Figure 1). Then we conducted hierarchical cluster analysis of the 245 proteins that provided an overall assessment of differential protein expression profile between CRC and adjacent normal mucosa (Figure 2).

**Figure 3:**
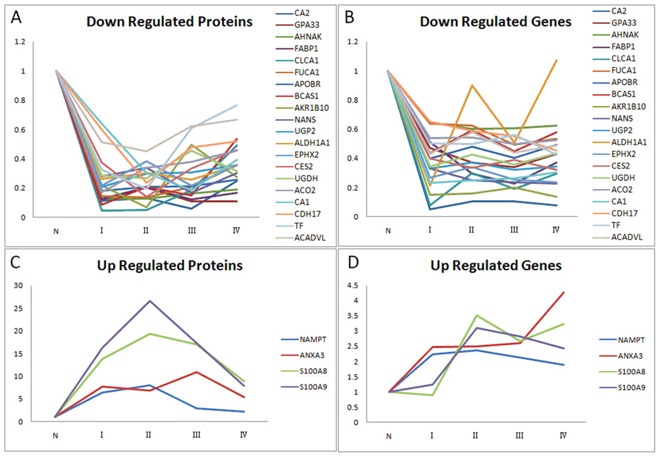
Relative ratios of 24 significantly differential proteins/genes amongst the 245 proteins/genes between paired CRC and adjacent normal mucosa independent of cancer stage **A.** Relative ratios of 20 down-regulated proteins among four stages of CRC and normal mucosa. **B.** Relative ratios of 20 down-regulated genes among four stages of CRC and normal mucosa. **C.** Relative ratios of 4 up-regulated proteins among four stages of CRC and normal mucosa. **D.** Relative ratios of 4 up-regulated genes among four stages of CRC and normal mucosa. Abbreviation: N, normal colonic mucosa.

Expression levels of 24 proteins (Table 2) amongst the 245 proteins were significantly different (p<0.05) between paired CRC and adjacent normal mucosa independent of cancer stage, which were confirmed in the validation set. Furthermore, compared with paired adjacent normal tissue, 4 proteins were up regulated in CRC, while 20 proteins were down regulated in CRC, so it was with their genes (Figure 3).

**Table 2:** Information of the final selected 24 proteins and their genes

SPN	Gene	Peptides (95%)	Protein express ratio(Ca/N)	Gene express ratio(Ca/N)	Protein express ratio validation(Ca/N)
P06702	**S100A9**	24	16.98204	2.754885	5.248075
P05109	S100A8	21	14.78627	2.849887	4.130475
P12429	**ANXA3**	28	7.751251	2.700335	3.311311
P43490	**NAMPT**	14	4.844349	2.195813	3.280953
P49748	**ACADVL**	33	0.561375	0.485372	0.809096
P02787	TF	58	0.473676	0.52579	0.337287
Q12864	**CDH17**	37	0.45606	0.565975	0.613762
P00915	CA1	39	0.382251	0.259204	0.30479
Q99798	ACO2	41	0.336688	0.515983	0.559758
O60701	UGDH	28	0.328707	0.383307	0.275423
O00748	**CES2**	16	0.325962	0.365841	0.222843
P34913	EPHX2	16	0.294428	0.282338	0.515229
P00352	**ALDH1A1**	30	0.293038	0.658422	0.159956
Q16851	UGP2	22	0.291184	0.339332	0.440555
Q9NR45	NANS	18	0.271187	0.248097	0.937562
O60218	AKR1B10	8	0.262586	0.177446	0.148594
O75363	BCAS1	11	0.24494	0.504075	0.319154
Q0VD83	APOBR	28	0.214114	0.43405	0.30479
P04066	FUCA1	10	0.209095	0.550799	0.15417
A8K7I4	**CLCA1**	52	0.194405	0.226036	0.376704
P07148	FABP1	52	0.154667	0.279866	0.636796
Q09666	AHNAK	276	0.1536	0.608789	0.275423
Q99795	GPA33	6	0.136646	0.367101	0.325087
P00918	CA2	19	0.136464	0.098199	0.272898

Abbreviations: N, normal colonic mucosa; Ca, colorectal cancer.

### Differential gene expression microarray analysis

We applied Affymetrix U133plus2.0 microarrays to detect differentially expressed genes between paired CRC and adjacent normal mucosa from another 52 patients (Table 1). The median age of the patients was 60.2 years old and about 57.7% were male.

In total, 39859 genes were identified in CRC and paired adjacent normal tissues, with 3083 genes having corresponding expressed proteins. Among the 3083 genes, there were 782 genes with expression level >1.5-fold change. As mentioned above, 245 of the 782 genes had corresponding proteins expressing differently by >1.5-fold and these genes had same regulation directions as their proteins did (Figure 1). Hierarchical cluster analysis of the 245 genes provided an overall assessment of differential gene expression profile between CRC and adjacent normal mucosa (Figure 2).

### Gene ontology enrichment analysis of the 245 differential proteins

**Figure 4:**
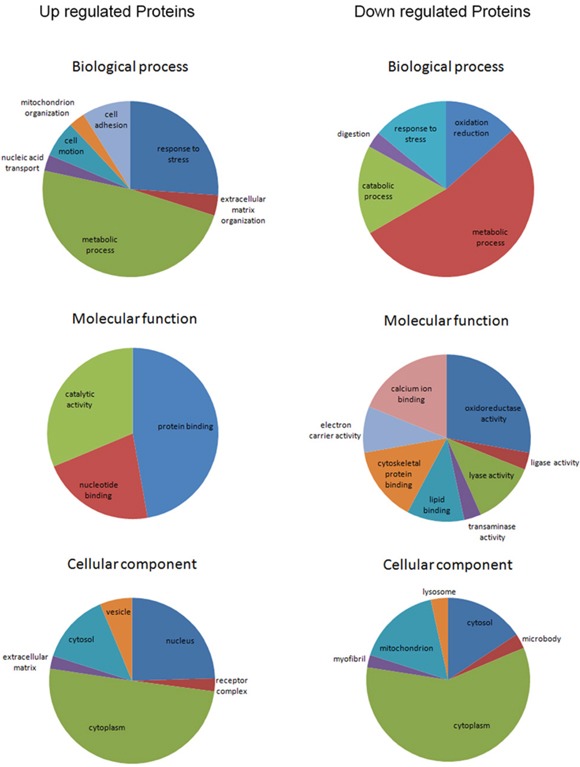
Gene ontology enrichment analyses for biological process, molecular function and cellular component of the 245 differential proteins (enrichment score>1.3)

To obtain a broad assessment of biological functions and cellular components of the 245 differential proteins, we performed gene ontology enrichment analysis using DAVID v6.7 Functional Annotation Clustering tools (enrichment score>1.3 or p<0.05, Figure 4). In general, the main biological processes in which up- and down-regulated proteins in CRC participated were metabolic process and response to stress. Up-regulated proteins were more involved in cell adhesion and motion, while down-regulated proteins were more associated with oxidation reduction reaction and catabolic process. As for molecular functions, a majority of up-regulated proteins clustered in protein binding. With regard to cellular components, both up- and down-regulated proteins were mainly located in cytoplasm. Additionally, up-regulated proteins were more likely to be located in nucleus and vesicles than down-regulated proteins.

### Verification of colorectal cancer potential biomarkers

**Figure 5:**
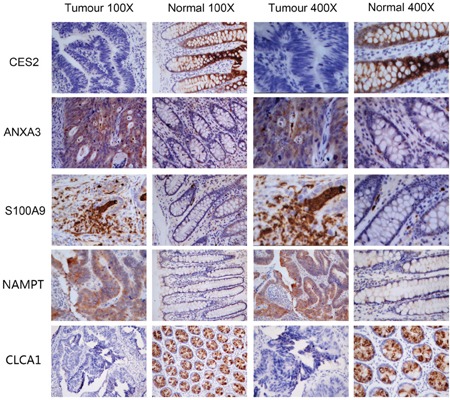
Immunohistochemistry images of S100A9, ANXA3, NAMPT, CES2 and CLCA1

To verify iTRAQ-MS data, we conducted IHC of 8 differential proteins in 18 paired samples, namely S100A9, ANXA3, NAMPT, ACADVL, CDH17, CES2, ALDH1A1 and CLCA1. Staining scores of 5 proteins (S100A9, ANXA3, NAMPT, CES2 and CLCA1) were significantly different between CRC and normal tissues (p<0.05, Table 3). In accordance with iTRAQ-MS results, S100A9, ANXA3 and NAMPT were up regulated in CRC, while CES2 and CLCA1 were down regulated in CRC. Figure 5 illustrated cellular localization of these five proteins.

**Table 3:** Results of immunohistochemistry

Proteins	Adjacent normal tissue		Cancer		p*
	Positive	Negative	Positive	Negative	
S100A9	0	18	11	7	<0.0001
ANXA3	7	11	16	2	0.004
NAMPT	1	17	14	4	<0.0001
ACADVL	11	7	8	10	0.250
CDH17	17	1	12	6	0.063
CES2	17	1	5	13	<0.0001
ALDH1A1	9	9	5	13	0.125
CLCA1	17	1	2	16	<0.0001

* paired chi-square test.

**Figure 6:**
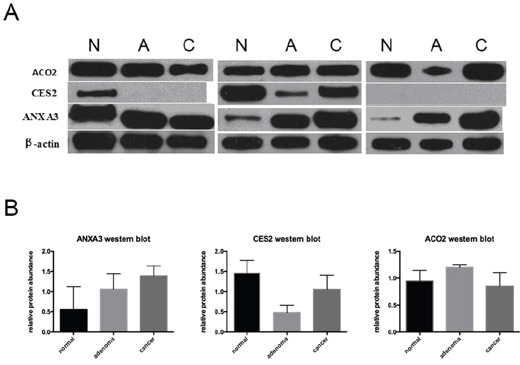
Western blot analysis of ACO2, CES2 and ANXA3 **A.** Western blot images of ACO2, CES2 and ANXA3. β-actin was used as loading control. **B.** Relative protein abundances determined by western blot (Mean±standard deviation). Abbreviations: N, normal colonic mucosa; A, adenoma; C, colorectal cancer.

Further validation was performed by western blot analysis of 3 representative proteins (i.e. ACO2, CES2 and ANXA3), using paired CRC, adenoma and normal fresh-frozen tissues of 3 patients (Table 4, Figure 6). The relative proteins abundances of normal tissues were not significantly different than cancer tissues because of limited sample size. However, the change tendencies of protein abundances in normal and paired CRC tissues were consistent with iTRAQ-MS ratios. Moreover, we observed that expression level of ANXA3 escalated in normal colorectal mucosa, adenoma and CRC tissues (Table 4, Figure 6B).

**Table 4:** Relative protein abundance of western blot analysis

	Normal tissue (mean±SD)	Adenoma (mean±SD)	Cancer (mean±SD)	p* (normal vs adenoma)	p* (normal vs cancer)
ACO2/β-actin	0.95±0.20	1.20±0.05	0.85±0.25	0.149	0.569
CES2/β-actin	1.47±0.30	0.48±0.18	1.05±0.36	0.006	0.129
ANXA3/β-actin	0.55±0.58	1.05±0.39	1.39±0.24	0.199	0.052

Abbreviations: SD, standard deviation.

* One-way ANOVA.

**Figure 7:**
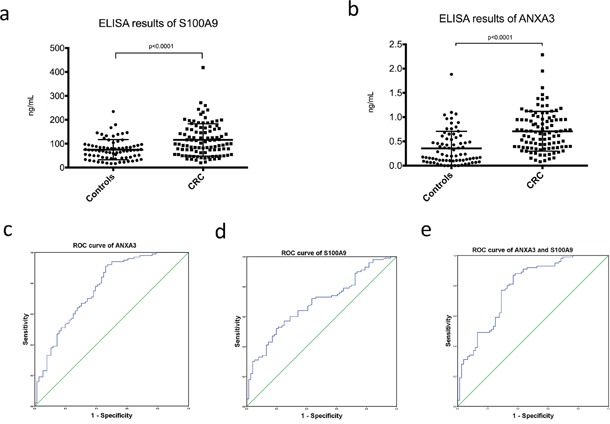
Serum ELISA results of S100A9 and ANXA3 in CRC patients and healthy controls **A.** Serum level of S100A9 in CRC was significantly higher than healthy controls (115.68±67.76 vs 74.84±42.11ng/mL, p<0.0001). **B.** Serum level of ANXA3 in CRC was significantly higher than healthy controls (0.71±0.41 vs 0.36±0.35ng/mL, p<0.0001). **C.** ROC curve of ANXA3 for CRC diagnosis (AUC=0.769, 95% CI=0.698-0.840, p<0.0001). **D.** ROC curve of S100A9 for CRC diagnosis (AUC=0.690, 95% CI=0.613-0.767, p<0.0001). **E.** ROC curve of ANXA3 combined with S100A9 for CRC diagnosis (AUC=0.788, 95% CI=0.720-0.857, p<0.0001). Abbreviations: ELISA, enzyme-linked immunosorbent assay; S100A9, S100 calcium-binding protein A9; ANXA3, annexin A3; CRC, colorectal cancer; ROC, receiver operator characteristic; AUC, area under curve; CI, confidence interval.

In addition, ELISA experiments were performed using serum samples of 100 CRC patients and 76 healthy controls. The results revealed that serum levels of S100A9 and ANXA3 in CRC patients were significantly higher than healthy controls (S100A9, 115.68±67.76 vs 74.84±42.11ng/mL, p<0.0001, Figure 7A; ANXA3, 0.71±0.41 vs 0.36±0.35ng/mL, p<0.0001, Figure 7B). Receiver operator characteristic (ROC) curves were drawn to evaluate the diagnostic value of ANXA3 and S100A9 for CRC. Area under curve (AUC) of ROC curve was measured and showed good prediction value (ANXA3, AUC=0.769, 95% confidence interval (CI)=0.698-0.840, p<0.0001, Figure 7C; S100A9, AUC=0.690, 95% CI=0.613-0.767, p<0.0001, Figure 7D; ANXA3 combined with S100A9, AUC=0.788, 95% CI=0.720-0.857, p<0.0001, Figure 7E).

### Inhibition of ANXA3 suppressed cell proliferation and aggressiveness

ANXA3-shRNA plasmid was transfected into SW620 cells to inhibit ANXA3 gene expression, obtaining transfectants named as SW620-ANXA3-shRNA. Meanwhile, a non-targeting plasmid was transfected in the same way as a negative control (SW620-NC).

**Figure 8:**
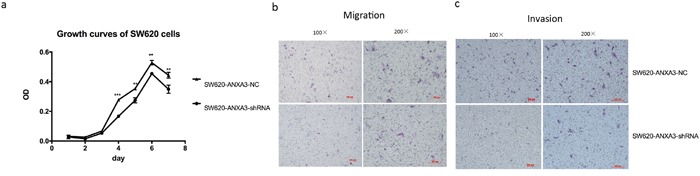
Inhibition of ANXA3 could suppress CRC cell proliferation and aggressiveness **A.** Cell growth curves revealed that from day 4 to day 7, the mean absorbance of SW620-ANXA3-shRNA was significantly lower than SW620-NC. **B.** The mean number of migrated SW620-ANXA3-shRNA cells in one visual field at a magnification of 200× was significantly fewer than SW620-NC (5.7±0.6 vs 18.7±2.5, p=0.001). **C.** The mean number of invaded SW620-ANXA3-shRNA cells in one visual field at a magnification of 200× was significantly fewer than SW620-NC (6.3±1.2 vs 14.3±1.2, p=0.001). Abbreviations: ANXA3, annexin A3; CRC, colorectal cancer; shRNA, small hairpin ribonucleic acid; NC, negative control. *p<0.05, **p<0.01, ***p<0.001.

Cell proliferation was evaluated by CCK-8 experiment. The results revealed that from day 4 to day 7, the mean absorbance of SW620-ANXA3-shRNA was significantly lower than SW620-NC. Cell growth curves indicated that inhibition of ANXA3 expression could significantly suppress SW620 cell proliferation (Figure 8A).

Transwell chamber was applied to evaluate cell migration and invasion abilities. We found that both migrated and invaded SW620-ANXA3-shRNA cells were significantly fewer than SW620-NC cells (mean number of migrated cells in one visual field at a magnification of 200×, 5.7±0.6 vs 18.7±2.5, p=0.001, Figure 8B; mean number of invaded cells in one visual field at a magnification of 200×, 6.3±1.2 vs 14.3±1.2, p=0.001, Figure 8C). Thus, we could conclude that inhibition of ANXA3 could reduce cell migration and invasion.

## DISCUSSION

In conclusion, iTRAQ-MS integrated with gene microarray of matched CRC and normal tissues identified over 3000 expressed proteins/genes, including 245 differentially expressed proteins that had same regulation directions as their corresponding genes. Gene ontology enrichment analysis showed that, unlike down-regulated proteins, up-regulated proteins were more involved in cell adhesion and motion. Besides, up-regulated proteins were more likely to be located in nucleus and vesicles. The final selected 24 proteins and their genes were expressed differently with statistical significance between paired CRC and adjacent normal mucosa independent of cancer stage, which were confirmed in the validation set. Further verification experiments with IHC confirmed differential expression levels of 5 proteins (S100A9, ANXA3, NAMPT, CES2 and CLCA1) between CRC and normal tissues. ELISA experiments validated diagnostic value of S100A9 and ANXA3. Cell experiments showed that inhibition of ANXA3 could suppress CRC cell proliferation and aggressiveness. In summary, our results suggested potential values of S100A9, ANXA3, NAMPT, CES2 and CLCA1 as biomarkers of CRC. Furthermore, ANXA3 was probably associated with CRC cell proliferation and aggressiveness.

S100A9, also known as calgranulin B, is a member of S100 protein family. As a calcium- and zinc-binding protein, S100A9 plays an important role in immune suppression of cancer. Previous studies have shown that, over-expression of S100A9 in tumor stroma can inhibit the differentiation of dendritic cells and macrophages and induce accumulation of myeloid-derived suppressor cells, thus is partly responsible for immune defects in cancer [9, 10]. Likewise, we observed that S100A9 were over expressed in CRC mesenchymal tissue.

ANXA3, a member of annexin family, is a calcium-dependent phospholipid-binding protein. Studies have demonstrated that ANXA3 is a potential biomarker for carcinogenesis, metastasis and resistance to chemotherapy in many cancers such as ovarian cancer [11], gastric cancer [12], breast cancer [13, 14] and liver carcinoma [15, 16]. But there is hardly any investigation of its role in CRC. Our results suggested that ANXA3 abundance increased stepwise in normal colorectal mucosa, adenoma and CRC tissues, indicating it being a candidate biomarker of colorectal carcinogenesis. Further *in vitro* experiments showed that inhibition of ANXA3 with shRNA plasmid could reduce CRC cell proliferation and aggressiveness, which indicated that ANXA3 might be a valuable therapeutic target of CRC. Further comprehensive study is expected.

NAMPT, also known as pre-B-cell colony-enhancing factor 1 (PBEF1) or visfatin, is an enzyme belonging to the family of glycosyltransferases. Nicotinamide adenine dinucleotide (NAD), one of the key metabolites needed for cell survival, has three main generating pathways including two salvage pathways. Compared to normal cells, tumor cells are more dependent on primary salvage pathway [17]. As the rate-limiting enzyme in primary salvage pathway, NAMPT expression level can greatly influence NAD metabolism and NAD dependent cellular processes in tumor cells [18]. Over-expression of NAMPT has been observed in many malignancies including colorectal, ovarian, breast, et al [19]. To date, a number of novel and potent inhibitors targeting NAMPT have been synthesized that have shown anti-tumor efficacy in tumor models *in vitro* and *in vivo* [20].

CES2, a member of alpha/beta fold hydrolase family, is an enzyme responsible for the hydrolysis of ester- and amide-bond-containing drugs. Its specific functions have not been elucidated yet. Studies have shown that some anti-cancer drugs such as 5-FU can induce CES2 expression and enhance cell-killing activity [21, 22]. We observed reduced expression of CES2 in CRC tissues compared to matched normal mucosa, which indicated that CES2 might be a negative biomarker of CRC risk.

CLCA1 belongs to the calcium sensitive chloride conductance protein family. Researchers have observed significantly lower expression of CLCA1 in colorectal cancer tissues compared with normal tissues. And low level of CLCA1 in CRC might predict disease relapse and poor prognosis [23, 24]. Our results were in concordance with previous studies. But the molecular mechanism how CLCA1 influences cancer cell proliferation and differentiation is still unknown.

Our study integrated high-throughput proteomics with genomics to identify candidate biomarkers of CRC. To our knowledge, except for our research, there has been only one study applying integrative proteomics with genomics to determine proteogenomic characterization of CRC so far [25]. Zhang and his colleagues conducted a study to perform integrated proteogenomic analyses of CRC, and identified five major proteomic subtypes that associated with clinical features [22]. With regard to our study, results of proteomics were highly consistent with that of genomics, showing 245 significantly differential proteins between CRC and paired normal mucosa. Further verification experiments with tissues indicated five proteins (S100A9, ANXA3, NAMPT, CES2 and CLCA1) to be potential biomarkers of CRC. But limitations such as small sample sizes and lack of experiments with body fluid existed in our study. Further large-scale prospective researches are warranted.

## MATERIALS AND METHODS

### Samples and patient characteristics

Paired CRC and adjacent normal tissues were surgically collected from 94 patients admitted in the Second Affiliated Hospital, Zhejiang University School of Medicine between 2004 and 2014 (Table 1a). Among the 94 paired samples, 76 pairs were fresh tissues and stored in refrigerator at −80°C, and 18 pairs were fixed in 10% buffered formalin and embedded in paraffin. Twenty-four pairs of all fresh frozen tissues were used for iTRAQ-LC-MS/MS, the remaining 52 pairs of fresh frozen tissues for gene microarray, while 18 pairs of formalin-fixed, paraffin-embedded tissues were applied for IHC. Besides, 3 samples among the 24 fresh frozen tissues used for iTRAQ-LC-MS/MS were also applied to perform western blot analysis, as well as their matched adenoma tissues. Table 1 listed patients' clinical characteristics of tissue samples.

Serum samples were collected and frozen at −80°C from 76 healthy controls and 100 CRC patients before treatment. The patients were admitted in the Second Affiliated Hospital, Zhejiang University School of Medicine between 2015 and 2016. All the serum samples were used for enzyme-linked immunosorbent assay (ELISA). Table 1b listed clinical characteristics of serum samples.

Written informed consents were obtained from all patients and Ethics Committee of the Second Affiliated Hospital, Zhejiang University School of Medicine approved this study.

### iTRAQ labeling of tissue extracts

Thawed tissues (24 CRC and 24 paired adjacent normal samples) were disrupted with ice-cold tissue protein lysis buffer (T-PER Reagent, added with phosphatase and protease inhibitor cocktail, Pierce, USA) in a glass homogenizor. Then tissue homogenates were centrifuged at 10,000g for 5 minutes at 4°C. Supernatants were collected and detected protein concentration by a BCA protein assay kit (ThermoFisher Scientific, USA). Equal amounts of proteins from 6 stage I CRC samples were pooled to one pooled-sample. Proteins from 6 stage II, 6 stage III, 6 stage IV and 24 adjacent normal samples were also pooled to one pooled-sample respectively. Each pooled-sample supernatant with 100 μg proteins were precipitated, reduced, alkylated and digested with trypsin. The five pooled-samples were labeled with one of the iTRAQ tags 113, 114, 116, 115, and 117, respectively with iTRAQ reagent kit (Applied Biosystems, CA). The 5 labeled samples were mixed, desalted with a Sep-Pak Vac C18 column (Waters, USA) and dried in a vacuum freeze dryer.

### Reversed phase liquid chromatography (RPLC) and MS/MS analysis

Dried samples were analyzed with a strong cation exchanger (SCX) liquid chromatography column. SCX fractionation was performed using a linear binary gradient of 2–25% buffer B (25% acetone (ACN), 10 mM KH_2_PO_4_, 350mM KCl, pH 2.6) in buffer A (25% ACN, 10 mM KH_2_PO_4_, pH 2.6) at a flow rate of 200 uL/min to separate the mixed iTRAQ-labeled samples into 10 fractions. Fractions were dried and resuspended in 5% ACN, 0.1% FA. Peptides were separated by a linear gradient formed by 5% ACN, 0.1% FA (mobile phase A) and 95% ACN, 0.1% FA (mobile phase B), from 5 to 40 of mobile phase B in 120 min at a flow rate of 300 nL/min. The MS analysis was performed on a TripleTOF 5600 system (AB SCIEX) in Information Dependent Mode. MS spectra were acquired across the mass range of 400–1500 m/z in high resolution mode (> 30 000) using 250 ms accumulation time per spectrum. A maximum of 15 precursors per cycle were chosen for fragmentation from each MS spectrum with 100 ms minimum accumulation time for each precursor and dynamic exclusion for 20 s. Tandem mass spectra were recorded in high sensitivity mode (resolution > 15 000) with rolling collision energy on and iTRAQ reagent collision energy adjustment on.

### Mass spectrometry data analysis

Protein identification was carried out using Protein Pilot 4.2 beta (ABSciex USA). The Paragon algorithm in ProteinPilot software served as the default search program with trypsin as the digestion agent and MMTS as a fixed modification of cysteine. Biological modifications were programmed in the algorithm. Each MS/MS spectrum was searched in the SWISSPROT database for Homo sapiens. Proteins were identified with Global FDR less than 5%.

### Gene microarray analysis

All samples were concurrently analyzed using Affymetrix U133plus2.0 microarrays. The extraction of RNA was performed following standard protocols provided by the manufacturers. For gene expression analysis, tumor and adjacent normal tissues were investigated using an Affymetrix U133plus2.0 microarray. Data were Acquired by GeneChip® Operating SoftwareVersion1.4. After quality checks, raw intensity data were Processed by quantile normalization with Robust Multi-Anlysis (RMA) to remove systematic bias using Affymetrix Expression Console Version 1.12.

### Proteomics data and gene expression data integration

MS protein quantification data and mRNA microarrays probe sets were annotated to gene symbols respectively. When multiple probe sets were mapped to a single gene symbols ID, the arithmetic mean of the all normalized probe sets was used for correlation computation. The proteins' fold change in CRC was calculated by mean values of fold changes in all the four stages of CRC.

### Western blotting

One-dimensional western blot analyses were performed utilizing standard procedures. Briefly, 10 μg of proteins were loaded and separated by standard 10% SDS-polyacrylamide gel electrophoresis (SDS-PAGE). Then proteins were transferred onto polyvinylidene fluoride (PVDF) membranes by electroblotting and probed with specific antibodies against ANXA3 (1:800, Abcam, ab33068), CES2 (1:1000, Abcam, ab184957), aconitase 2 (ACO2, 1:1000, Abcam, ab12910) andβ-actin(1:10000, HuaAn Biotechnology, M1210-2). Immunoreactive bands were detected by chemiluminescence using corresponding horseradish peroxidase (HRP)-conjugated secondary antibodies and enhanced chemiluminescence (ECL) detection reagents.

### IHC and semi-quantitative analysis

The avidin–biotin–peroxidase complex method was used for immunostaining. In brief, paraffin-embedded blocks were sectioned at about 5μm thickness. Slides were baked at 60°C overnight, deparaffinized with xylene and rehydrated using graded ethanol series. After antigen retrieval process, the tissue slides were treated with 3% hydrogen peroxide for 15 min to quench endogenous peroxidase activity. After 15 min of preincubation in 10% normal goat serum to prevent nonspecific staining, the sections were incubated with the diluted primary antibody at 37°C for 2 h, followed sequentially by peroxidase-polymer labeled rabbit anti-mouse secondary antibody. Then sites of peroxidase activity were demonstrated with diaminobenzidine. Finally sections were counterstained with hematoxylin and differentiated in hydrochloric acid alcohol, followed by dehydrated and mounted.

Primary antibodies used in IHC analysis included S100A9 (1:1000, Abcam, ab92507), ANXA3(1:150, Abcam, ab33068), NAMPT (1:250, Abcam, ab109210), acyl-CoA dehydrogenase, very long chain (ACADVL, 1:400, Abcam, ab155138), cadherin 17 (CDH17, 1:1000, Abcam, ab109220), CES2(1:500, Abcam, ab184957), acetaldehyde dehydrogenase 1A1 (ALDH1A1, 1:100, Abcam, ab52492) and CLCA1(1:1000, Abcam, ab180851).

Staining expression was assessed by two independent pathologists based on both intensity and distribution of positive cells. Staining intensity was graded as follows: 0=absent, 1=low, 2=moderate and 3=high. Staining distribution was determined by percentage of positive cells (0, <5% positive cells; 1, 5-25% positive cells; 2, 26-50% positive cells; 3, >50% positive cells). The two scores were added by each other and divided by 2 to obtain the final score, which was categorized as negative for score <2 and positive for score ≥2.

### ELISA

Serum levels of selected proteins in CRC patients and healthy volunteers were evaluated using commercially available sandwich ELISA kit according to the manufacturers' protocol (S100A9, CUSABIO, CSB-E11834h; ANXA3, Cloud-Clone Corp., SEE786Hu). All the samples and standards were detected in duplicate.

### Cell culture and ANXA3-shRNA plasmid transfection

SW620 cells were cultured in Leibovitz L-15 medium at 37°C in 5% CO_2_. Culture medium was supplemented with 10% fetal bovine serum (FBS, Gibco), 100 U/mL penicillin and 100mg/mL streptomycin. ANXA3-small hairpin RNA (shRNA) plasmid was bought from Sangon Biotech and transfected into SW620 cells using Lipofectamine 3000 reagent (Invitrogen). Meanwhile, a non-targeting plasmid was used as a negative control.

### Cell proliferation assay

Cell Counting Kit-8 (CCK-8, KeyGen) was used to measure cell proliferation. Assays were performed according to the manufacturers' protocol. Cell proliferation was measured by optimal density (OD) value of 450 nm. The mean and SD were calculated from 3 independent experiments.

### Cell migration and invasion assay

Cell migration was evaluated using transwell chamber with an 8μm-pore filter membrane (Corning Inc.). About 2×10^5^ Cells in serum-free medium were seeded in the upper chamber, while conditioned medium with 20% FBS was added to the lower chamber. The chambers were incubated for 48 hours in 37°C incubator. Non-migrated cells in the upper chamber were removed with cotton swabs, whereas migrated cells on the underside of filter membrane were fixed in 4% (v/v) paraformaldehyde and stained with crystal violet. Cell invasion was evaluated using matrigel coated 8μm-pore transwell chamber (Corning Inc.). The procedures of cell invasion assay were identical to cell migration. The migrated/invaded cells were counted by light microscopy. The migration/invasion ability was measured by mean cell number of five visual fields at a magnification of 200×. The experiments were carried out in triplicate.

### Statistical analysis

SPSS Statistics 20.0 (IBM, Armonk, NY, USA) was used to perform statistical analysis. Statistical tests were two sided and p < 0.05 was considered statistically significant. Chi-square test was applied to compare categorical variables. Two-tailed Student's t-test and one-way ANOVA were used to compare quantitative data.

## Abbreviations

CRC: colorectal cancer
iTRAQ-MS: isobaric tags with related and absolute quantitation labeling mass spectrometry
IHC: immunohistochemistry
WHO: World Health Organization
TCGA: the Cancer Genome Atlas
S100A9: S100 calcium-binding protein A9
ANXA3: annexin A3
NAMPT: nicotinamide phosphoribosyltransferase
CES2: carboxylesterase 2
CLCA1: calcium activated chloride channel A1
RPLC: reversed phase liquid chromatography
SCX: strong cation exchanger
CAN: acetone
SDS-PAGE: SDS-polyacrylamide gel electrophoresis
PVDF: polyvinylidene fluoride
ACO2: aconitase 2
HRP: horseradish peroxidase
ECL: enhanced chemiluminescence
ELISA: enzyme-linked immunosorbent assay
CCK-8: Cell Counting Kit-8
PBEF1: pre-B-cell colony-enhancing factor 1
NAD: nicotinamide adenine dinucleotide
ROC: receiver operator characteristic
AUC: area under curve
CI: confidence interval
shRNA: small hairpin ribonucleic acid
NC: negative control.
